# The Sensitivity and Specificity of Loop-Mediated Isothermal Amplification (LAMP) Assay for Tuberculosis Diagnosis in Adults with Chronic Cough in Malawi

**DOI:** 10.1371/journal.pone.0155101

**Published:** 2016-05-12

**Authors:** Marriott Nliwasa, Peter MacPherson, Palesa Chisala, Mercy Kamdolozi, McEwen Khundi, Kruger Kaswaswa, Mphatso Mwapasa, Chisomo Msefula, Hojoon Sohn, Clare Flach, Elizabeth L. Corbett

**Affiliations:** 1 Helse Nord Tuberculosis Initiative, Department of Microbiology, College of Medicine, Blantyre, Malawi; 2 Malawi-Liverpool-Welcome Trust Clinical Research Programme, Blantyre, Malawi; 3 Clinical Research Department, London School of Hygiene & Tropical Medicine (LSHTM), London, United Kingdom; 4 Department of Public Health and Policy, University of Liverpool, Liverpool, United Kingdom; 5 Department of Clinical Sciences, Liverpool School of Tropical Medicine, Liverpool, United Kingdom; 6 Department of Surgery, College of Medicine, Blantyre, Malawi; 7 Department of Epidemiology, Biostatistics & Occupational Health, McGill University, Montreal, Canada; 8 Department of Infectious Disease Epidemiology, London School of Hygiene & Tropical Medicine (LSHTM), London, United Kingdom; University College London, UNITED KINGDOM

## Abstract

**Background:**

Current tuberculosis diagnostics lack sensitivity, and are expensive. Highly accurate, rapid and cheaper diagnostic tests are required for point of care use in low resource settings with high HIV prevalence.

**Objective:**

To investigate the sensitivity and specificity, and cost of loop-mediated isothermal amplification (LAMP) assay for tuberculosis diagnosis in adults with chronic cough compared to Xpert® MTB/RIF, fluorescence smear microscopy.

**Methods:**

Between October 2013 and March 2014, consecutive adults at a primary care clinic were screened for cough, offered HIV testing and assessed for tuberculosis using LAMP, Xpert^®^ MTB/RIF and fluorescence smear microscopy. Sensitivity and specificity (with culture as reference standard), and costs were estimated.

**Results:**

Of 273 adults recruited, 44.3% (121/273) were HIV-positive and 19.4% (53/273) had bacteriogically confirmed tuberculosis. The sensitivity of LAMP compared to culture was 65.0% (95% CI: 48.3% to 79.4%) with 100% (95% CI: 98.0% to 100%) specificity. The sensitivity of Xpert^®^ MTB/RIF (77.5%, 95% CI: 61.5% to 89.2%) was similar to that of LAMP, p = 0.132. The sensitivity of concentrated fluorescence smear microscopy with routine double reading (87.5%, 95% CI: 73.2% to 95.8%) was higher than that of LAMP, p = 0.020. All three tests had high specificity. The lowest cost per test of LAMP was at batch size of 14 samples (US$ 9.98); this was lower than Xpert® MTB/RIF (US$ 13.38) but higher than fluorescence smear microscopy (US$ 0.65).

**Conclusion:**

The sensitivity of LAMP was similar to Xpert^®^ MTB/RIF but lower than fluorescence smear microscopy; all three tests had high specificity. These findings support the Malawi policy that recommends a combination of fluorescence smear microscopy and Xpert® MTB/RIF prioritised for people living with HIV, already found to be smear-negative, or being considered for retreatment of tuberculosis.

## Introduction

Early diagnosis of tuberculosis with highly accurate, rapid point of care tests is a key component of the WHO’s End Tuberculosis Strategy [1]. The Xpert^®^ MTB/RIF is being rolled out in low-and middle- income countries and is now a major player in tuberculosis prevention and care [2, 3].

The loop-mediated isothermal amplification (LAMP) assay for diagnosing tuberculosis is a new test with a number of similarities to Xpert® MTB/RIF: it is mainly intended for use with sputum specimens, and is a nucleic acid amplification test [4]. LAMP is less fully automated than the Xpert® MTB/RIF system, providing both potential advantages (higher peak throughput, and lower capital costs) and disadvantages (greater operator complexity, potential for cross-contamination and error in interpreting visual fluorescent readout) [5]. LAMP is being considered as a potential replacement for smear microscopy in settings with low burden of multi-drug resistant tuberculosis, with potential cost-savings compared to Xpert® MTB/RIF in peripheral laboratories with high volume of samples, and countries not eligible for low income country access prices [6]. A recent meta-analysis estimated a sensitivity of 80% and specificity of 96% for LAMP on sputum specimens compared to culture, but included studies were mostly from countries with low burden of HIV [7].

Combined HIV testing and counselling for adults with symptoms of tuberculosis identifies people in need of ART [8]. Although an international policy recommendation for some time, HIV testing and counselling for adults with symptoms of tuberculosis has not been fully implemented within the primary health care system in sub-Saharan Africa [9–11].

The objective of this study was to investigate the sensitivity and specificity, and costing estimate of LAMP for diagnosing tuberculosis in a low resource setting with high prevalence of HIV. The study also investigates the importance of HIV testing for adults with symptoms of tuberculosis and provides sensitivity and specificity and costing estimates of Xpert® MTB/RIF and concentrated fluorescence smear microscopy.

## Methods

### Study design and setting

This diagnostic evaluation study was conducted at Ndirande Health Centre, Blantyre, Malawi. The health centre serves a high density urban area of Blantyre city, with population prevalence of HIV and tuberculosis of 18.5% and 0.9%, respectively [12, 13]. Tuberculosis diagnostic services (with fluorescence smear microscopy and Xpert^®^ MTB/RIF for smear negative HIV positive individuals) are routinely provided at the health centre [14]. Chest radiography and culture are only available by referral to the city’s central hospital.

### Study participants

Between October 2013 and March 2014, consecutive patients attending the outpatient department of Ndirande Health Centre were informed about the study while in the waiting area. Trained research assistants undertook an eligibility assessment with all facility attenders who were willing to participate in the study. Patients were eligible if they had chronic cough (≥2 weeks), were at least 15 years old and were residing in Blantyre city. Participants were excluded if they could not submit sputum, or were taking tuberculosis treatment, or had reported that they had been treated for tuberculosis in the past.

Ethics approval was obtained from the College of Medicine of Malawi Research and Ethics Committee. Approval number P.06/13/1397. Written informed consent was obtained from all participants. For participants under the age of 18 years, informed written or witnessed thumbprint participant assent and parental or guardian consent were required for recruitment.

### Participant interview

Research assistants interviewed all attending eligible individuals to record socio-demographic information and prior access to HIV care services.

All participants were asked to submit two spot sputum samples for tuberculosis diagnosis. Samples were collected in an outside, well-ventilated space of the health centre.

Participants were offered counselling and HIV testing using OraQuick ADVANCE Rapid HIV 1/2 Antibody Test (OraSure Technologies, Bethlehem, Pennsylvania, USA) on oral fluid. Determine HIV 1/2 test (Abbott Laboratories, Abbott Park, IL) and Uni-Gold™ Recombigen® HIV-1/2 (Trinity Biotech, Wicklow, Ireland) were used to confirm HIV positive results.

### Linkage to TB and HIV care and vital status at 2 months

Participants diagnosed with tuberculosis were registered for treatment at the health centre. Treatment outcomes were ascertained by extracting data from tuberculosis treatment registers.

HIV positive participants were referred to an HIV care clinic based within the health centre for assessment for antiretroviral therapy (ART) initiation.

A follow-up study visit was organised for all participants at 2 months to assess vital status and linkage to tuberculosis and HIV care. Participants who were unable to come to the clinic were followed-up with a phone call or a home visit by study field workers.

### Case definitions for tuberculosis

A bacteriologically confirmed tuberculosis case was defined as being a participant with a positive result by smear microscopy, Xpert^®^ MTB/RIF or culture [15]. Participants started on tuberculosis treatment without bacteriological confirmation were classified as having ‘clinically diagnosed tuberculosis’.

### Laboratory methods

**Fig 1:**
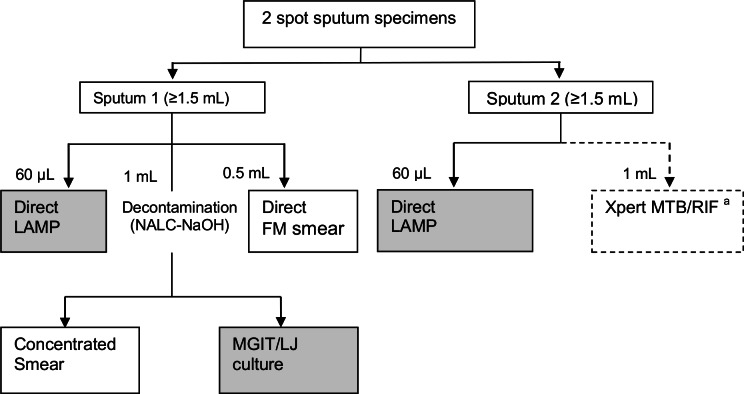
Laboratory workflow. Abbreviations: LAMP = Loop-mediated Isothermal Amplification assay; FM = fluorescence microscopy; MGIT = Mycobacteria Growth Indicator Tube; LJ = Lowenstein Jensen media; NALC-NaOH = n-acetyl-L-cysteine (NALC)—sodium hydroxide (NaOH). ^a^ Preference was given to investigations for ‘sputum 1’ in case of inadequate volume or salivary specimen of one or more samples, since culture results were the predefined reference standard.

Sputum testing for tuberculosis used LAMP as an index test, and culture as the reference standard. Sputum samples were additionally examined by fluorescence smear microscopy and Xpert^®^ MTB/RIF (Fig 1).

### LAMP assay procedure

LAMP was performed by a trained technician in Ndirande Health Centre’s microscopy laboratory. The technician had passed a proficiency test after one week training. Two spot sputum samples were run on LAMP for each participant (Fig 1).

A semi-automated LAMP (Eiken Chemical Co., Ltd.) assay, described previously [4, 16], was used. This LAMP assay joins manual sputum processing and DNA extraction with isothermal amplification and visual readout of results in a fluorescence detector. Assays were run according to manufacturer’s instructions and results were recorded qualitatively; a positive result when there was ‘green fluorescent light emitted’ and a negative result when there was ‘no fluorescent light emitted’ [17].

### Xpert® MTB/RIF, smear microscopy and culture

After the LAMP assay, the two spot sputum samples from each participant were transferred to a specialist laboratory (College of Medicine, Blantyre) for concentrated smear microscopy and culture (1st sample) and Xpert^®^ MTB/RIF (2nd sample). Where sample volume was suboptimal, preference was given to the predetermined reference standard (culture).

Smear microscopy used auramine with Primo Star iLED™ (Carl Zeiss Microimaging, Oberkochen, Germany). Culture used BD BACTEC™ MGIT™ 960 (MGIT) and Lowenstein Jensen (LJ). Smear and culture results were interpreted by different technicians, both without reference to LAMP results.

Species identification used MPT 64 antigen test (MGIT TBc Identification test, Becton Dickinson) plus microscopic cording; if either rapid identification test was negative then identification was based on compatible growth characteristics at various temperatures (37°C, room temperature and 45°C) and growth on p-nitrobenzoate LJ slopes.

### Statistical methods

The primary outcome was the sensitivity and specificity of LAMP compared to culture. We calculated that 237 samples were required assuming a prevalence of culture confirmed tuberculosis of 30% [18] and a sensitivity of LAMP on culture positive specimens of 88.2% [16], accepting a 95% confidence interval (CI) of ± 3.75% width [19]. We conducted an exploratory analysis of sensitivity and specificity of LAMP by HIV and smear status.

The following were other pre-planned analyses: 1) Sensitivity and specificity of each of Xpert® MTB/RIF and fluorescence smear microscopy (with culture as reference standard); 2) Yield of undiagnosed HIV; 3) Linkage to HIV and tuberculosis care; and 4) Vital status at 2 months.

Results of each of LAMP, Xpert MTB/RIF and fluorescence smear microscopy were coded as “positive” or “negative”. In the exploratory comparison of the sensitivity of LAMP by smear and HIV status, we used Fisher’s exact test. For comparing sensitivities of Xpert MTB/RIF and fluorescence smear microscopy with LAMP, we considered this as a paired study design and used McNemar’s test. Data analysis used Stata 13.1 (StataCorp LP, College Station, Texas) and R version 3.2.3 (package “DTComPair”).

### Cost analysis

Cost per test for each of LAMP, fluorescence smear microscopy and Xpert® MTB/RIF was determined. For each of the diagnostic tests, complete cost data were measured considering the following; assay costs, labour, overhead costs, building costs, maintenance costs, running costs of building, management and supervision costs and costs associated with equipment consumables and chemicals [20]. A range of batch size scenarios were observed and resource usage during each of the test runs was recorded. The cost per test was expressed in United States dollars (US$); prices available in the local currency (Malawian Kwacha) were converted into US$ based on the average United Nations operational exchange rate in 2014 [21].

## Results

### Recruitment

**Fig 2:**
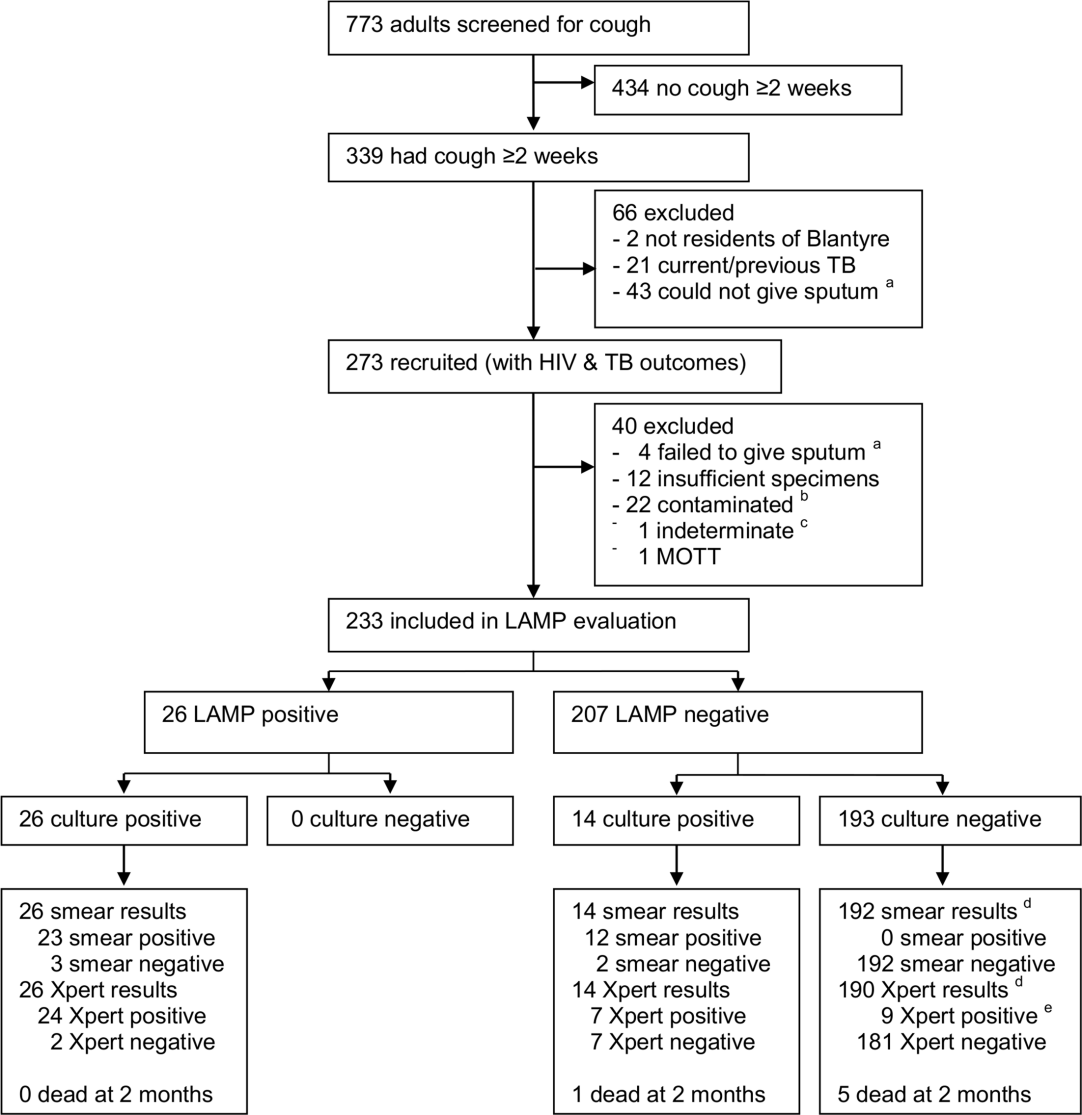
Recruitment of participants and distribution of TB diagnosis results. Abbreviations: MOTT = *Mycobacteria* other than tuberculosis; Xpert = Xpert MTB/RIF. ^a^ Participants could not give sputum despite having cough ≥ 2 weeks. ^b^ All contaminated samples were smear negative. ^c^ indeterminate result if culture negative and smear positive. ^d^ Missing results: 1 fluorescence smear result and 3 Xpert MTB/Rif result. ^e^ participants with culture- and smear- negative results had ‘MTB detected low’ results on Xpert® MTB/RIF.

A total of 773 adults (≥ 15 years) were screened for cough. There were 339 adults who met the eligibility criterion of chronic cough, of whom 273 were recruited. Reasons for exclusion are summarised in Fig 2. Baseline characteristics, tuberculosis and HIV diagnostic outcomes are presented in Table 1. Overall, 52% of the participants were female. Participants were mostly young adults with a median age of 32 years (interquartile range [IQR], 25 to 41).

**Table 1:** Baseline characteristics and tuberculosis and HIV diagnostic outcomes.

Characteristic	HIV-positive	HIV-negative	P-value
	N (%)	N (%)	
Number ^a^	121	130	
Sex, female	64 (52.9)	56 (43.1)	0.120
Age (years), median (IQR)^ ^	35 (30–43)	30 (22–41)	<0.001
15–24 years	10 (8.3)	40 (31.7)	<0.001
25–34 years	41 (33.9)	43 (33.6)	
35–44 years	45 (37.2)	20 (15.6)	
≥45 years	25 (20.7)	25 (19.2)	
Body mass index, median (IQR)	20 (18–22)	22 (19–24)	0.003
Access to HIV testing and ART			
HIV positive on ART	34 (28.0)		
HIV positive not on ART	34 (28.0)		
New HIV positive	53 (44.0)		
Confirmatory HIV testing ^b^	63/87 (72.4)		
CD 4 count results ^b^			
No CD4 count result	34/87 (30.1)		
0–199	29/87 (33.3)		
200–349	18/87 (20.7)		
350–400	4/87 (4.6)		
≤500	2/87 (2.3)		
CD 4 count (cells/mm3) median (IQR) ^b^	175 (102–274)		
Vital status at 2 months			
Dead	5 (4.3)	2 (1.5)	0.298
Alive	105 (89.7)	111 (85.4)	
Lost to follow-up	11 (9.1)	17 (13.1)	
All TB cases ^c^	24 (19.8)	24 (18.5)	0.782
Smear + /culture + TB	12 (9.9)	17 (13.1)	0.434
Smear—/culture + TB	4 (3.3)	2 (1.4)	0.360
Xpert® MTB/RIF + only	6 (5.0)	4 (3.1)	0.446
Clinically diagnosed TB ^d^	2 (1.7)	1 (0.8)	0.520
TB treated	19 (15.7)	21 (16.2)	1.000
TB treatment outcome			
Cured/Completed	15/19 (78.9)	15/21 (71.4)	0.271
Dead	3/19 (15.7)	1/21 (4.8)	
Lost to follow-up	1/19 (5.3)	3/21 (14.3)	
Transferred out	0/19 (0.0)	2/21 (9.5)	

Abbreviations: ART = antiretroviral therapy; TB = tuberculosis; + = positive;— = negative

^a^ Participants who refused HIV test and are not shown, 22 (8.1%)

^b^ Participants with newly diagnosed HIV or were not previously on ART were referred for confirmatory HIV testing (blood testing with Determine and UniGold) and assessment of CD 4 cell count in routine care

^c^ 8 tuberculosis cases were diagnosed among participants with unknown HIV status, 5 of these were started on treatment, none died

^d^ Participants who were culture negative and smear negative and were started on TB treatment based on clinical decision

### Yield of tuberculosis

A total of 56/273 (20.5%) participants were diagnosed with tuberculosis, 53/273 (19.4%) had bacteriologically-confirmed tuberculosis and 3/273 (1.1%) had clinically-diagnosed tuberculosis. Tuberculosis cases are shown by HIV test and method of diagnosis (i.e. smear, culture, and Xpert® MTB/RIF) in Table 1 and Fig 2.

### Yield of previously undiagnosed HIV

Overall, 92% (251/273) of participants accepted HIV testing. The prevalence of HIV was 48.2% (121/251). Of those HIV positive, 43.8% (53/121) were newly diagnosed and 28% (34/121) were known to be HIV positive but not yet on ART (Table 1).

### Sensitivity and specificity of LAMP

There were 233/273 (85.3%) participants who were included in the evaluation of LAMP (Fig 2). Reasons for exclusion were missing or insufficient specimen; contaminated culture results; smear-positive culture-negative results; or culture positive results due to *Mycobacterium* other than tuberculosis (MOTT) (Fig 1). Agreement between LAMP results on first and second samples was almost perfect (kappa = 0.94) (Fig 1). For those included in LAMP evaluation, MGIT culture results were available for all, 233. No participant had an LJ-positive MGIT-negative specimen. Therefore, the reference standard was entirely derived from the MGIT results.

The overall sensitivity of LAMP compared to culture was 65.0% (95% CI: 48.3% to 79.4%) and the specificity was 100% (95% CI: 98.0% to 100%) (Table 2). Sensitivity and specificity of LAMP did not differ significantly by HIV or smear status although power was limited for this sub-analysis (Table 2). The sensitivity of LAMP was 66.7% (95% CI: 38.4% to 88.2%) among HIV positive participants compared to 52.6% (95% CI: 28.9% to 75.6%) among HIV negative participants, p = 1.000. The sensitivity of LAMP on smear-positive culture-positive specimens was 65.7% (95% CI: 47.8% to 80.9%) compared to 60.0% (95% CI: 14.7% to 94.7%) on smear-negative culture-positive specimens, p = 0.495.

**Table 2:** Sensitivity and specificity of LAMP (compared to MGIT) by HIV status and smear status (n = 233).

	Sensitivity			Specificity
	Overall	Smear positive and culture positive	Smear negative and culture positive	
**Overall (n = 233)** ^a^				
Correct-no/total no	26/40 (65.0%)	23/35 (65.7%)	3/5 (60.0%)	193/193 (100%)
95% CI	(48.3%– 79.4%)	(47.8%– 80.9%)	(14.7%– 94.7%)	(98.0%– 100%)
**HIV Positive (n = 102)**				
Correct-no/total no	10/15 (66.7%)	8/12 (66.7%)	2/3 (66.7%)	87/87 (100%)
95% CI	(38.4%– 88.2%)	(34.9%– 90.0%)	(9.4%– 99.2%)	(95.9%– 100%)
**HIV Negative (n = 112)**				
Correct-no/total no	10/19 (52.6%)	9/17 (52.9%)	1/2 (50.0%)	93/93 (100%)
95% CI	(28.9%– 75.6%)	(27.8%– 77.0%)	(1.3%– 98.7%)	(96.1%– 100%)

^a^ Includes 19 samples from participants with unknown HIV status

### Sensitivity and specificity of Xpert® MTB/RIF and smear microscopy

The sensitivity of concentrated fluorescence smear microscopy (87.5%, 95% CI: 73.2% to 95.8%) was higher than that of LAMP (65.0%, 95% CI: 48.3% to 79.4%), p = 0.020. The sensitivity of Xpert® MTB/RIF (77.5%, 95% CI: 61.5% to 89.2%) was similar to that of LAMP, p = 0.132. All three tests had high specificity: Xpert® MTB/RIF, 95.3% (95% CI: 91.2% to 97.8%); concentrated fluorescence smear microscopy, 100% (95% CI: 98.1% to 100%); and LAMP, 100% (95% CI: 98.0% to 100%) (Table 3 and S2 Table).

**Table 3:** Diagnostic accuracy of LAMP, Xpert® MTB/RIF and fluorescence smear microscopy and cost per test at different batch sizes (n = 233).

	LAMP	Xpert MTB/RIF ^a^	FM microscopy
**Sensitivity**			
Correct-no/total no	26/40 (65.0%)	31/40 (77.5%)	35/40 (87.5%)
95% CI	(48.3%– 79.4%)	(61.5%– 89.2%)	(73.2%– 95.8%)
**Specificity**			
Correct-no/total no	193/193 (100%)	(181/190) 95.3%	192/192 (100%)
95% CI	(98.1%– 100%)	(91.2%– 97.8%)	(98.1%– 100%)
**Cost per test**			
2 tests per batch	16.88	14.50	-
4 tests per batch	13.38	13.01	1.42
10 tests per batch	10.65	13.51	1.23
14 tests per batch	9.98	13.38	0.65

Abbreviations: LAMP = loop-mediated isothermal amplification assay; FM = fluorescence microscopy

^a^ 4 module Xpert® MTB/RIF was used

### Cost per test of LAMP, Xpert® MTB/RIF and smear microscopy

The cost per test of Xpert® MTB/RIF was more favourable than LAMP for batch sizes less than four (Table 3). A batch of 14 samples was less costly on LAMP (US$ 9.98) than on Xpert® MTB/RIF (US$ 13.38). Fluorescence microscopy had the lowest cost per test at all batch sizes; the cost per test at a batch of size of 14 samples was US$ 0.65.

### Linkage to HIV/tuberculosis care and vital status at 2 months

Of the 56/273 (20.5%) participants diagnosed with tuberculosis, 45/56 (80.4%) were started on treatment by 2 months (Table 1). Pre-treatment loss to follow-up was 19.6% (11/56) reasons were 1 withdrew from the study and 10 could not be traced by both phone call and home visit.

Of the 121/273 (44.3%) participants who were HIV positive, 87/121 (71.9%) participants had newly diagnosed HIV or were known to be HIV positive but not yet on ART (Table 1). There was a loss to participation down the continuum of HIV care; 72% (63/87) of participants with newly diagnosed HIV or those known to be HIV positive but not yet on ART had confirmatory HIV testing in routine care and at 2 months follow-up only 60.9% (53/87) had been assessed for ART eligibility using CD 4 cell count (Table 1). All participants assessed had low CD 4 cell count (175/μL,IQR, 102–274) and were eligible for ART [22].

There were 242/273 (88.6%) participants with vital status ascertained at 2 months, of whom 7/242 (2.9%) had died. Four more deaths occurred among participants treated for tuberculosis before they completed treatment (6 months) (Table 1).

## Discussion

The main finding of this study is that the sensitivity of LAMP was similar to that of Xpert® MTB/RIF but lower than that of concentrated fluorescence smear microscopy; all three tests had high specificity. Based on these data, we recommend that LAMP should not be used to replace fluorescence smear microscopy or Xpert® MTB/RIF in peripheral laboratories of countries like Malawi that are eligible for the “FIND-access price” for Xpert® MTB/RIF machines and cartridges [6].

**Fig 3:**
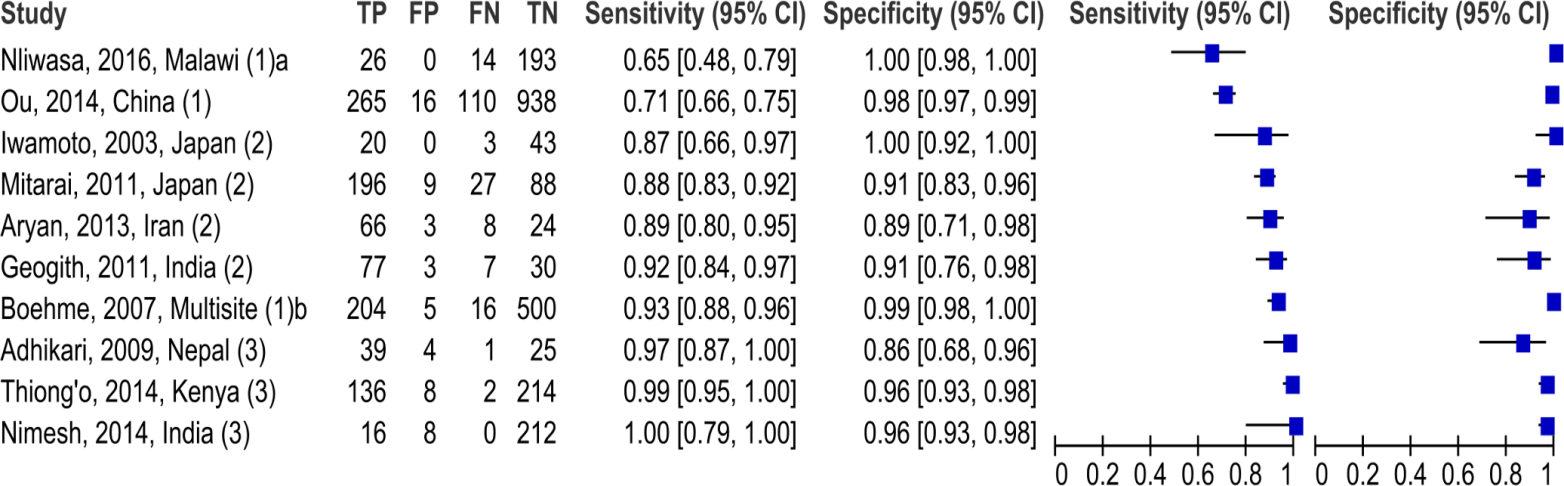
Studies of accuracy of LAMP for diagnosis of tuberculosis. Abbreviations: TP = true positive; FP = false positive; FN = false negative; TN = true negative. Study setting: ^1^ Primary care; ^2^ Hospital; ^3^ University or national reference laboratory. ^a^ Refers to findings of this study in Malawi.^b^ Study sites were in Peru, Bangladesh, Tanzania.

In this study, LAMP had a sensitivity of 65% and specificity of 100%. Compared to other studies at primary care level, the sensitivity of LAMP is similar to that reported in China [17] but lower than in Tanzania, Peru and Bangladesh [16] (Fig 3). The sensitivity of LAMP in this study was lower than that reported from studies in hospitals [4, 23–25] and in university or national reference laboratories [26–28] (Fig 3).

In this study, fluorescence microscopy had a high sensitivity of 87.5% and specificity of 99.5% and was the cheapest. Microscopy is highly operator-dependant and so our results are unlikely to be generalizable to laboratories with less expertise in microscopy or less emphasis on quality assurance. Xpert® MTB/RIF had sensitivity of 77.5% and specificity of 95.3%; much lower than expected, sensitivity, 98.2% and specificity, 99.2% (2). Performance of Xpert® MTB/RIF in our laboratory may have been affected by the prioritisation of smear microscopy and culture (Fig 1).

Pre-treatment loss to follow-up among tuberculosis patients was 19.6% and falls in range of 6 to 30% reported from studies in Africa [29]. Pre-treatment loss to follow-up is due to: individual factors like male sex, older age, being diagnosed with smear-negative but culture-positive tuberculosis; and system related factors including delays in receiving results, long waiting times in health services, the need for repeated visits, and geographical location of the tuberculosis laboratory (regional versus local) [29].

Linkage to HIV care was suboptimal, with only 60.9% of participants in need of ART successfully assessed for ART initiation at 2 months. This suboptimal linkage to assessment for ART has also been reported among adults identified in the community, 56.3% [30] and in a primary care clinic 54.6% [31] and is influenced by many factors including the individual’s social support networks, attitude of health workers and the organisation of patient flow in health facilities [32].

There are a number of limitations to this study. LAMP was conducted at primary care level while Xpert® MTB/RIF and fluorescence microscopy were conducted at a research laboratory with more rigorous laboratory standards. Due to the small sample size, we were unable to investigate sensitivity and specificity of LAMP by HIV or smear status. The sensitivity of Xpert® MTB/RIF was affected by the prioritisation of microscopy and culture over Xpert® MTB/RIF. Diagnosis of tuberculosis was mainly based on sputum. Participants who were not able to submit sputum were not actively investigated for tuberculosis e.g. with chest radiography or sputum induction.

## Conclusion

The sensitivity of LAMP was similar Xpert® MTB/RIF but lower than concentrated fluorescence smear microscopy; all three tests had high specificity. LAMP should not replace fluorescence smear microscopy or Xpert® MTB/RIF. These findings support the Malawi policy that recommends a combination of fluorescence microscopy and Xpert® MTB/RIF prioritised for people living with HIV, already found to be smear-negative, or being considered for retreatment of tuberculosis.

## Supporting Information

S1 Table LAMP minimal dataset (CSV)

S2 Table Additional measures of diagnostic accuracy (DOCX)

## References

[1] World Health Organisation. End TB Strategy: Global strategy and targets for tuberculosis prevention, care and control after 2015 2015 [cited 2015 14 October 2015]. Available: http://www.who.int/tb/post2015_strategy/en/.

[2] Boehme CC, Nabeta P, Hillemann D, Nicol MP, Shenai S, Krapp F, et al Rapid molecular detection of tuberculosis and rifampin resistance. New England Journal of Medicine. 2010;363(11):1005–15. 10.1056/NEJMoa0907847 20825313 PMC2947799

[3] World Health Organisation. Global Tuberculosis Report 2015. Geneva: World Health Organization; 2015.

[4] Mitarai S, Okumura M, Toyota E, Yoshiyama T, Aono A, Sejimo A, et al Evaluation of a simple loop-mediated isothermal amplification test kit for the diagnosis of tuberculosis. Int J Tuberc Lung Dis. 2011;15(9):1211–7, i. Epub 2011/09/29. 10.5588/ijtld.10.0629 . 21943848

[5] UNITAID. Tuberculosis diagnostic technology landscape: semi-annual update 2012 [cited 2015 24 August 2015]. Available: https://globalhealthmarketdynamics.wordpress.com/resources/tuberculosis-diagnostic-technology-landscape/.

[6] Foundation for innovative new diagnostics. Price for Xpert® MTB/RIF and FIND country list 2013 [cited 2015 07 October 2015]. Available: http://www.finddiagnostics.org/about/what_we_do/successes/find-negotiated-prices/xpert_mtb_rif.html.

[7] Yuan LY, Li Y, Wang M, Ke ZQ, Xu WZ. Rapid and effective diagnosis of pulmonary tuberculosis with novel and sensitive loop-mediated isothermal amplification (LAMP) assay in clinical samples: a meta-analysis. J Infect Chemother. 2014;20(2):86–92. Epub 2014/01/28. 10.1016/j.jiac.2013.07.003 . 24462417

[8] Munthali L, Mwaungulu JN, Munthali K, Bowie C, Crampin AC. Using tuberculosis suspects to identify patients eligible for antiretroviral treatment. International Journal of Tuberculosis & Lung Disease. 2006;10(2):199–202. . 16499261

[9] MacPherson P, Lalloo DG, Choko AT, Mann GH, Squire SB, Mwale D, et al Suboptimal patterns of provider initiated HIV testing and counselling, antiretroviral therapy eligibility assessment and referral in primary health clinic attendees in Blantyre, Malawi. Trop Med Int Health. 2012;17(4):507–17. Epub 2012/02/03. 10.1111/j.1365-3156.2011.02946.x ; PubMed Central PMCID: PMCPmc3378506. 22296187 PMC3378506

[10] Van Rie A, Clouse K, Hanrahan C, Selibas K, Sanne I, Williams S, et al High uptake of systematic HIV counseling and testing and TB symptom screening at a primary care clinic in South Africa. PLoS One. 2014;9(9):e105428 Epub 2014/10/01. 10.1371/journal.pone.0105428 ; PubMed Central PMCID: PMCPmc4182031. 25268851 PMC4182031

[11] World Health Organization. WHO policy on collaborative TB/HIV activities: Guidelines for national programmes and other stakeholders. 2012. 23586124

[12] Choko AT, Desmond N, Webb EL, Chavula K, Napierala-Mavedzenge S, Gaydos CA, et al The uptake and accuracy of oral kits for HIV self-testing in high HIV prevalence setting: a cross-sectional feasibility study in Blantyre, Malawi. PLoS Med. 2011;8(10):e1001102 Epub 2011/10/13. 10.1371/journal.pmed.1001102 ; PubMed Central PMCID: PMCPmc3186813. 21990966 PMC3186813

[13] National Tuberculosis Control programme. Malawi Nationwide Tuberculosis Prevalence Survey. 45^th^ Union World Conference on Lung Health. Barcelona, Spain: 2014.

[14] Ministry of Health. National tuberculosis control programme manual, 7th edition—2012. 2012 [cited 2015 22 March 2015]. Available: http://www.medcol.mw/globalhealth/uploads/MalawianTBguidelines2012.pdf.

[15] World Health Organisation. Definitions and reporting framework for tuberculosis Geneva: WHO: 2013.

[16] Boehme CC, Nabeta P, Henostroza G, Raqib R, Rahim Z, Gerhardt M, et al Operational feasibility of using loop-mediated isothermal amplification for diagnosis of pulmonary tuberculosis in microscopy centers of developing countries. J Clin Microbiol. 2007;45(6):1936–40. Epub 2007/03/30. 10.1128/jcm.02352-06 ; PubMed Central PMCID: PMCPmc1933042. 17392443 PMC1933042

[17] Ou X, Li Q, Xia H, Pang Y, Wang S, Zhao B, et al Diagnostic accuracy of the PURE-LAMP test for pulmonary tuberculosis at the county-level laboratory in China. PLoS One. 2014;9(5):e94544 Epub 2014/05/03. 10.1371/journal.pone.0094544 ; PubMed Central PMCID: PMCPmc4006777. 24788724 PMC4006777

[18] Munyati SS, Dhoba T, Makanza ED, Mungofa S, Wellington M, Mutsvangwa J, et al Chronic cough in primary health care attendees, Harare, Zimbabwe: diagnosis and impact of HIV infection. Clinical Infectious Diseases. 2005;40(12):1818–27. . 15909272 10.1086/429912

[19] Buderer NM. Statistical methodology: I. Incorporating the prevalence of disease into the sample size calculation for sensitivity and specificity. Acad Emerg Med. 1996;3(9):895–900. Epub 1996/09/01. . 8870764 10.1111/j.1553-2712.1996.tb03538.x

[20] Sohn H, Sinthuwattanawibool C, Rienthong S, Varma JK. Fluorescence microscopy is less expensive than Ziehl-Neelsen microscopy in Thailand. Int J Tuberc Lung Dis. 2009;13(2):266–8. Epub 2009/01/17. . 19146758

[21] United Nations. UN Operational Rates of Exchange [cited 2015 14 August 2015]. Available: http://treasury.un.org/operationalrates/OperationalRates.aspx.

[22] INSIGHT START Study Group. Initiation of Antiretroviral Therapy in Early Asymptomatic HIV Infection. N Engl J Med. 2015 Epub 2015/07/21. 10.1056/NEJMoa1506816 . 26192873 PMC4569751

[23] Iwamoto T, Sonobe T, Hayashi K. Loop-mediated isothermal amplification for direct detection of Mycobacterium tuberculosis complex, M. avium, and M. intracellulare in sputum samples. J Clin Microbiol. 2003;41(6):2616–22. Epub 2003/06/07. ; PubMed Central PMCID: PMCPmc156570. 12791888 10.1128/JCM.41.6.2616-2622.2003 PMC156570

[24] Aryan E, Makvandi M, Farajzadeh A, Huygen K, Alvandi AH, Gouya MM, et al Clinical value of IS6110-based loop-mediated isothermal amplification for detection of Mycobacterium tuberculosis complex in respiratory specimens. J Infect. 2013;66(6):487–93. Epub 2013/03/08. 10.1016/j.jinf.2013.02.005 . 23466595

[25] Geojith G, Dhanasekaran S, Chandran SP, Kenneth J. Efficacy of loop mediated isothermal amplification (LAMP) assay for the laboratory identification of Mycobacterium tuberculosis isolates in a resource limited setting. J Microbiol Methods. 2011;84(1):71–3. Epub 2010/11/05. 10.1016/j.mimet.2010.10.015 . 21047534

[26] Adhikari BR, Pandey BD, Ghimire P, Shrestha B, Khadka M, Yoda T, et al Loop-mediated isothermal amplification (LAMP) for the direct detection of human pulmonary infections with environmental (nontuberculosis) mycobacteria. Jpn J Infect Dis. 2009;62(3):212–4. Epub 2009/05/27. . 19468184

[27] Nimesh M, Joon D, Varma-Basil M, Saluja D. Development and clinical evaluation of sdaA loop mediated isothermal amplification assay for the detection of M. tuberculosis with an approach to prevent carry-over contamination. J Clin Microbiol. 2014 Epub 2014/05/03. 10.1128/jcm.00907-14 . 24789191 PMC4097723

[28] Thiong’o SW. Evaluation of loop-mediated isothermal amplification as a point-of-care diagnostic tool for mycobacterium tuberculosis: University of Nairobi; 2014.

[29] MacPherson P, Houben RM, Glynn JR, Corbett EL, Kranzer K. Pre-treatment loss to follow-up in tuberculosis patients in low- and lower-middle-income countries and high-burden countries: a systematic review and meta-analysis. Bull World Health Organ. 2014;92(2):126–38. Epub 2014/03/14. 10.2471/blt.13.124800 24623906 PMC3949536

[30] Choko AT, MacPherson P, Webb EL, Willey BA, Feasy H, Sambakunsi R, et al Uptake, Accuracy, Safety, and Linkage into Care over Two Years of Promoting Annual Self-Testing for HIV in Blantyre, Malawi: A Community-Based Prospective Study. PLoS Med. 2015;12(9):e1001873 Epub 2015/09/09. 10.1371/journal.pmed.1001873 26348035 PMC4562710

[31] MacPherson P, Corbett EL, Makombe SD, van Oosterhout JJ, Manda E, Choko AT, et al Determinants and consequences of failure of linkage to antiretroviral therapy at primary care level in Blantyre, Malawi: a prospective cohort study. PLoS One. 2012;7(9):e44794 Epub 2012/09/18. 10.1371/journal.pone.0044794 22984560 PMC3439373

[32] MacPherson P, MacPherson EE, Mwale D, Bertel Squire S, Makombe SD, Corbett EL, et al Barriers and facilitators to linkage to ART in primary care: a qualitative study of patients and providers in Blantyre, Malawi. J Int AIDS Soc. 2012;15(2):18020 Epub 2013/01/23. 10.7448/ias.15.2.18020 ; PubMed Central PMCID: PMCPmc3535694. 23336700 PMC3535694

