# Supplementary material for: The Sensitivity and Specificity of Loop-Mediated Isothermal Amplification (LAMP) Assay for Tuberculosis Diagnosis in Adults with Chronic Cough in Malawi
Source: PLoS One. 2016 May 12;11(5):e0155101. doi: 10.1371/journal.pone.0155101 (PMC4865214; doi:10.1371/journal.pone.0155101)
Supplement: S2 Table — (DOCX) [file pone.0155101.s002.docx]

| **Test** | **TP** | **FN** | **TN** | **FP** | **Measure** | **% (95% CI)** |
| --- | --- | --- | --- | --- | --- | --- |
| LAMP | 26 | 14 | 193 | 0 | Sensitivity | 65.0% (48.3% – 79.4%) |
|  |  |  |  |  | Specificity | 100% (98.0% – 100%) |
|  |  |  |  |  | Positive predictive value | 100% (86.8% - 100%) |
|  |  |  |  |  | Negative predictive value | 93.2% (88.9% – 96.3%) |
|  |  |  |  |  | Diagnostic accuracy ^a^ | 94.0% (90.1% – 96.7%) |
| Xpert MTB/Rif | 31 | 9 | 181 | 9 | Sensitivity | 77.5% (61.5% – 89.2%) |
|  |  |  |  |  | Specificity | 95.3% (91.2% – 97.8%) |
|  |  |  |  |  | Positive predictive value | 77.5% (61.5% - 89.2%) |
|  |  |  |  |  | Negative predictive value | 95.3% (91.2% – 97.8%) |
|  |  |  |  |  | Diagnostic accuracy ^a^ | 92.2% (87.9% – 95.3%) |
| Fluorescence smear microscopy | 35 | 5 | 192 | 0 | Sensitivity | 87.5% (73.2% – 95.8%) |
|  |  |  |  |  | Specificity | 100% (98.1% – 100%) |
|  |  |  |  |  | Positive predictive value | 100% (90.0% - 100%) |
|  |  |  |  |  | Negative predictive value | 97.5% (94.2% – 99.2%) |
|  |  |  |  |  | Diagnostic accuracy ^a^ | 97.8% (95.0% - 99.3%) |

**S2 Table. Additional measures of diagnostic accuracy**

^a^ A global measure of diagnostic accuracy, the so called diagnostic accuracy (effectiveness) is expressed as a proportion of correctly classified subjects (TP+TN) among all subjects (TP+TN+FP+FN).
